# Natural Ingredient-Based Polymeric Nanoparticles for Cancer Treatment

**DOI:** 10.3390/molecules25163620

**Published:** 2020-08-09

**Authors:** Ka Hong Wong, Aiping Lu, Xiaoyu Chen, Zhijun Yang

**Affiliations:** 1 School of Chinese Medicine, Hong Kong Baptist University, Hong Kong 999077, China; 16483081@life.hkbu.edu.hk (K.H.W.); aipinglu@hkbu.edu.hk (A.L.); cxyu2016@hkbu.edu.hk (X.C.); 2 Changshu Research Institute, Hong Kong Baptist University, Changshu Economic and Technological Development (CETD) Zone, Changshu 215500, China

**Keywords:** drug delivery system, natural materials, nanoparticles, polymers, cancer therapy

## Abstract

Cancer is a global health challenge. There are drawbacks to conventional chemotherapy such as poor bioavailability, development of drug resistance and severe side effects. Novel drug delivery system may be an alternative to optimize therapeutic effects. When such systems consist of natural materials, they offer important advantages: they are usually highly biocompatible, biodegradable, nontoxic and nonimmunogenic. Furthermore, natural materials can be easily modified for conjugation with a wide range of therapeutic agents and targeting ligands, according to the therapeutic purpose. This article reviews different natural ingredients and their applications in drug delivery systems for cancer therapy. Firstly, an overview of the polysaccharides and protein-based polymers that have been extensively investigated for drug delivery are described. Secondly, recent advances in using various natural ingredient-based polymeric nanoparticles for cancer therapy are reviewed. The characteristics of these delivery systems are summarized, followed by a discussion of future development and clinical potential. This review aims to summarize current knowledge and provide a basis for developing effective tailor-made formulations for cancer therapy in the future.

## 1. Introduction

Cancer is still a major health challenge in the world. It has been estimated that there will be over 1.8 million new cases and more than 0.6 million cancer deaths in the United States in 2020 [1]. Cancer treatment depends on the cancer type, tumor stage, biomarkers and the patient’s general health. Therapies include surgery, radiation therapy, chemotherapy, targeted therapy and immunotherapy [2,3,4,5,6]. Among them, chemotherapy predominates due to its high cytotoxicity against cancer cells. However, conventional chemotherapy has serious drawbacks: low response, drug resistance, severe side effects, poor bioavailability and nonspecific distribution [6]. Reducing these side effects would greatly enhance its efficacy and value as a therapeutic approach.

As we learn more about the pathology and biology of cancer, targeted therapy and immunotherapy have the potential to play a more significant role in cancer treatment. Drug delivery systems (DDS) show various advantages as compared to conventional chemotherapy. They can deliver the drug to a specific tumor site, facilitate drug clearance from the circulatory and immune system, alter the physicochemical properties of drugs, reduce the dose needed and control the drug release; such characteristics give DDS great potential for cancer therapy [7,8]. A wide range of ingredients such as polymers, lipids, proteins and metallic particles have been employed as drug delivery carriers to encapsulate various therapeutic molecules. According to the origin of the carriers, it can be classified into natural or synthetic materials. The natural materials are usually abundant in nature and inexpensive. They are highly biocompatible, biodegradable, nontoxic and nonimmunogenic. Furthermore, functional groups on the natural material molecules allow them to be easily modified for conjugation with drug molecules or targeting ligands, or the formation of copolymers [9]. Additionally, they may carry specific protein binding sites and other biochemical signals that may assist in tissue engineering or localized delivery [10]. The benefits of natural materials make them attractive and promising in developing DDS. Although there are disadvantages, including batch-to-batch variations and structural complexity, the advantages outweigh their shortcomings [10].

In this review, we focus on the applications of different natural ingredients as drug carriers to deliver anticancer agents. First, the characteristics of the natural materials are reviewed because such properties determine the performance of DDS. In the second part, various natural ingredient-based DDSs used in cancer therapy are discussed. The advantages and drawbacks of the mentioned systems are summarized, followed by a discussion of future development and clinical potential in the last part. This review aims to provide a perspective for developing effective tailor-made DDSs for future clinical applications.

## 2. Natural Polymers

Natural polymers come from animals, plants, bacteria and fungi. There are two main types: polysaccharides and protein-based polymers. Both have been extensively investigated for drug delivery. Both can form scaffolds as a viable extracellular matrix (ECM). In this way, targeted drug delivery can be achieved with high loading efficiency and minimal invasive behavior [11,12]. Furthermore, the functional groups present on the polymer backbones can be modified easily for functionalization. The use of polysaccharides and polymers for drug delivery has shown promising results [9,13]. In this section, the most frequently used natural polymers for drug delivery are discussed.

### 2.1. Polysaccharides

**Figure 1:**
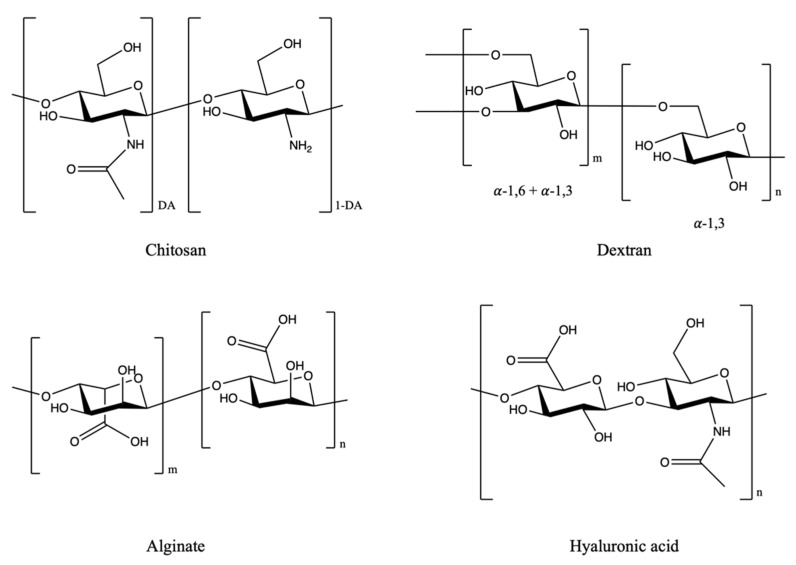
Chemical structures of polysaccharides used as drug carriers (DA: degree of deacetylation; m,n: no. of units).

Polysaccharides are long chains of polymeric carbohydrates composed of simple monosaccharide (sugar) repeating units linked by glycosidic bonds (Figure 1). Polysaccharides consist of more than ten simple sugar units. In nature, polysaccharides can be obtained and derived from animals (e.g., chitosan, chondroitin), plants (e.g., pectin, guar gum, mannan), algals (e.g., alginate) and microbes (e.g., dextran, xanthan gum) [14]. Nontoxicity, biocompatibility and good stability make them be good candidates for drug delivery [14,15]. Among them, chitosan, dextran, alginates and hyaluronic acid are the most frequently used materials for drug delivery.

#### 2.1.1. Chitosan

Chitosan is a linear cationic amino polysaccharide obtained by deacetylation of chitin (degree of deacetylation over 50%). It is composed of β-d-glucosamine and *N*-acetyl-d-glucosamine units by 1, 4 glycosidic linkages. Chitosan has outstanding biocompatibility, mucoadhesivity, antibacterial and antifungal properties and nontoxicity [16,17,18]. It can be degraded by enzymes to oligomers and monomers [16]. However, the poor solubility of chitosan in a neutral or high pH environment restricts its biological applications. This problem can be solved by modifying the functional groups on the backbone; other of its biological activities can also be regulated by this method [19]. After modification, chitosan can be functionalized as DDSs, e.g., as hydrogels, nanoparticles, microspheres and micelles for cancer treatment.

#### 2.1.2. Hyaluronic Acid

Hyaluronic acid (also called hyaluronan) is a linear anionic glycosaminoglycan composed of glucuronic acid and N-acetylglucosamine units by β-1,4 and β-1,3 glycosidic linkages. Hyaluronic acid can bind with several cell surface receptors such as CD44, which is always found in tumor ECM and is associated with tumor progress [20]. As a result, hyaluronic acid can be utilized to develop DDSs for targeted therapy. Hyaluronidases are the enzymes expressed in the body to degrade hyaluronic acid. Hyaluronidases are highly expressed in tumor microenvironments [21,22]. Therefore, controlled drug release of hyaluronic acid DDSs can be achieved when hyaluronic acid is degraded by hyaluronidase [23]. However, hyaluronic acid also plays a role in tumor cell proliferation and angiogenesis with complex bioactivities. It should pay more attention when developing DDS by using hyaluronic acid as a carrier for cancer treatment [20].

#### 2.1.3. Alginates

Alginates are linear anionic polymers composed of irregular repeating units of β-d-mannuronic acid and α-L-guluronic with 1,4 glycosidic linkages. Alginic acid is insoluble in water but its monovalent salt is soluble; sodium alginate is the most common salt form [24]. Alginate can form a gel under mild reaction conditions (pH, temperature, etc.) without using toxic solvents. The quality of the gel depends on the gelation rate which is affected by ions present in the solution, temperature and the natural chemical structure of the alginate [24,25]. After the formation of the gel, water molecules can still migrate inside the gel matrix. One problem is that, as the alginates are obtained from seaweeds, impurities such as heavy metals and protein may be entrapped in the matrix of alginate molecules [26]. Nevertheless, alginates have great potential in developing DDSs, particularly hydrogels, for cancer treatment.

#### 2.1.4. Dextran

Dextran is composed of branched glucan by the α-1,6 linkage between d-glucose units; side chains may be present at the α-1,2, α-1,3, or α-1,4 positions. The molecular structure of dextran is highly related to its source. Molecular weight and degree of branching may affect its biological activities [27]. Dextran has decent water solubility and flexibility for functional modifications. Dextran can stabilize the DDSs or drug conjugates and enhance the bioavailability of a drug after administration without significant toxicity [28]. Therefore, it and its derivatives are suitable for developing as drug carriers such as micelles and hydrogels.

### 2.2. Protein-Based Polymers

The basic elements of proteins are amino acids. The amino acids are linked via peptide bonds, while the three-dimensional structure is stabilized by hydrophobic interaction, hydrogen bonding, disulfide bonding and salt bridges. Protein-based polymers are usually derived from natural tissues. They generally have good biocompatibility and biodegradability. They can undergo natural degradation so that the accumulation of byproducts after drug delivery is minimal [29]. Among proteins, collagen, albumin and gelatin are the most frequently used materials for drug delivery.

#### 2.2.1. Collagen

Collagen is the most abundant protein in vertebrates. Type I collagen is the dominant type in ECM. Due to its high biocompatibility and biodegradability, collagen can be utilized for tissue engineering and processed into various types of DDS. However, collagen can be dissociated during isolation and purification. Furthermore, natural degradation leads collagen formulations to suffer from stability problems. To solve these issues and improve its mechanical properties, collagen is often crosslinked with other natural-based polymers or synthetic polymers [30,31]. By choosing appropriate crosslinking materials and methods, the properties of collagen can be altered according to the therapeutic needs. With modifications, stability and the release profile can be controlled.

#### 2.2.2. Gelatin

Gelatin is a natural, biocompatible and biodegradable biopolymer derived from the hydrolysis of animal collagen. Gelatin can dissolve in water rapidly; this solubility may compromise its effectiveness for long-term drug delivery when employed as a drug vehicle. Similar to collagen, the mechanical and physiochemical properties of gelatin can be modified by coupling with different crosslinkers and targeting ligands, thereby altering the release profile of gelatin [32]. Moreover, gelatin can be employed for the preparation of thermoreversible gels, which broadens the applications of gelatin in pharmaceutical sciences [33].

#### 2.2.3. Albumin

The different types of albumin include human serum albumin (HSA), bovine serum albumin (BSA), rat serum albumin (RSA) and ovalbumin (OSA). Among them, HSA and BSA are the most frequently used for DDS development. They are highly soluble proteins with no toxicity and low immunogenicity. There are many binding sites on the protein molecules; therefore, a drug can be conjugated with albumin via covalent bonding or just be adsorbed on its surface [34,35]. Albumin can also be employed as a drug carrier for cancer therapeutics. It was reported that albumin-based nanoparticles were able to penetrate the blood-brain barrier (BBB). By modifying the surface of albumin nanoparticles with cell-penetrating peptides, the BBB penetration could be enhanced [36].

## 3. Drug Delivery Systems for Cancer Treatment

**Figure 2:**
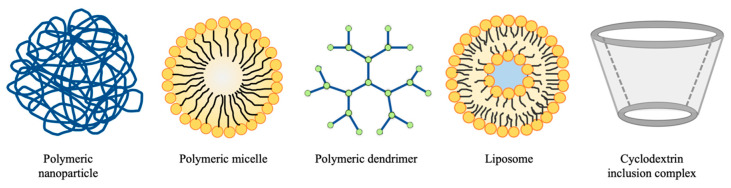
Various types of drug delivery systems (DDS) consisting of natural ingredients.

The use of DDS is a strategy for overcoming the disadvantages of chemotherapy for cancer treatment [7,8]. In the last section, different types of natural polymers were reviewed. All of them can be employed as drug carriers to encapsulate therapeutics agents to form a DDS (Figure 2). The characteristics of the DDS depend on its components, surface properties and architecture. Various natural ingredients can be chosen according to the therapeutic purposes. In some cases, more than one natural ingredient can be used to form the DDS [37]. In this section, various types of natural polymer-based nanoparticles designed for targeted cancer therapy are discussed.

### 3.1. Nanoparticles

Polymeric nanoparticles (NPs) with diameters within the range from 10 to 1000 nm are ideal for drug delivery [38]. Natural polymer-based nanoparticles for cancer diagnosis and therapy have already been reported. Employing natural material particles for drug delivery will add extra biocompatibility to the whole delivery system. By adjusting the properties of polymers or modifying the surface of nanoparticles with various targeting ligands, targeted delivery and controlled drug release can be achieved [39].

#### 3.1.1. Chitosan-Based Nanoparticles for Cancer Therapy

Chitosan and its derivatives have been widely applied in anticancer drug delivery (Table 1). Anticancer agents can be encapsulated by the nanoparticles or attached directly. Chitosan ascorbate nanoparticles were reported to show inhibition effects on cervical cancer [40]. Sekar et al. first prepared chitosan ascorbate from chitosan and ascorbic acid by salification. Then, nanoparticles were obtained by ionotropic gelation using pentasodium tripolyphosphate (TPP) as a crosslinker under acidic conditions. The particle size of the nanoparticles was about 170 nm, which could be controlled by adjusting the concentration of ascorbic acid. The nanoparticles showed antioxidative ability. They lowered the viability of cervical cancer cells (HeLa cells) but did not change the survival rate of normal cells (human fibroblasts WI-38). In this study, it showed the potential of the chitosan ascorbate nanoparticles to serve as nanocarriers for cancer-targeted drug delivery [40]. However, more data, including in vivo study, are required to verify the functions of this formulation. The surface of chitosan-based nanoparticles can be modified for multiple purposes. Nascimento et al. reported epidermal growth factor receptor (EGFR)-targeted chitosan nanoparticles for delivery of siRNA to treat non-small cell lung cancer (NSCLC) [41]. EGFR is overexpressed in the NSCLC microenvironment, which can serve as a target site for drug delivery. In the study, EGFR binding peptide and PEG2000 were conjugated with chitosan to form derivatives. Then, the chitosan derivatives were complexed with Mad2 siRNA to form the targeted nanoparticles. Two types of chitosan with different molecular weights were employed. Nanoparticles consisting of lower molecular weight (50 kDa) chitosan gave smaller particle size as compared to that of the higher molecular weight (90 kDa) chitosan. Both formulations showed nearly 100% encapsulation efficiency. Both EGFR-targeted chitosan nanoparticles showed higher selective uptake by NSCLC cells (A549 cells). The encapsulated siRNA successfully knocked down Mad2 (a mitotic checkpoint component), resulting in massive cancer cell death by apoptosis. A lower molecular weight chitosan formulation showed better activity than a higher molecular weight formulation [41]. The lower molecular weight formulation was further investigated in cisplatin-sensitive and -resistant mice models. The targeted formulation showed stronger inhibition of tumor growth when compared to a nontargeted formulation. The effect in a platinum-resistant model was even better [42]. Chitosan-based nanoparticles can also be employed for small molecule delivery. Boroujeni et al. developed a folate-modified chitosan nanoparticle to encapsulate curcumin for breast cancer treatment [43]. Folate can bind to the folate receptors overexpressed in cancer cells. Therefore, it can be employed as a targeting ligand for DDSs. Curcumin was released from the nanoparticles in a sustained manner over one week. The release rate in acidic conditions was faster due to the protonation of amine groups on the chitosan backbone, causing the polymer matrix to swell. A similar phenomenon was reported by Vivek et al. [44]. This formulation showed a better therapeutic effect than free curcumin in breast cancer cells (MCF-7 cells), and the bare nanoparticles had no toxicity to noncancerous cells (L929 cells) [43]. Chitosan nanoparticles could also deliver doxorubicin for hepatic cancer treatment. Tian et al. developed a chitosan nanoparticle with glycyrrhetinic acid (GA) modification, which was able to bind with rat hepatocytes and the GA receptors on human hepatic cells for liver targeting [45,46]. The GA-modified chitosan nanoparticles showed strong cellular uptake by human hepatic carcinoma cells (QGY-7703 cells). Loading doxorubicin with the nanoparticle formulation, doxorubicin showed stronger cytotoxicity towards QGY-7703 cells and showed better performance in inhibition of tumor growth in H22 cell-bearing mice, as compared to the free drug [45]. Niu et al. developed a thermosensitive and pH-sensitive liposome with cell-penetrating peptide modification to encapsulate doxorubicin for triple-negative breast cancer treatment. First, poly(N-vinylcaprolactam) (PNVCL) was attached to the chitosan backbone. PNVCL is a biocompatibility polymer that exhibits a reversible phase transition from hydrophilic to hydrophobic when the temperature is raised. Chitosan was responsible for the production of pH-sensitive DDS because its solubility slightly increases in acidic environments. The conjugate was further modified with cell-penetrating peptide (sequence: RLYMRYYSPTTRRYG) to enhance the permeability of DDS. Finally, the copolymers self-assembled into nanoparticles in aqueous solution and loaded doxorubicin into the hydrophobic core. Drug release from the nanoparticles was accelerated in acidic and hyperpyrexic conditions. The formulation selectively enhanced the cellular uptake of doxorubicin by MCF-7 cells but not healthy human umbilical vein endothelial (HUVEC) cells. The cell-penetrating peptide facilitated the formulation to accumulate at the tumor site after administration. The DDS demonstrated stronger tumor inhibition in the xenograft mouse model with less off-target effects and lower systemic toxicity than treatment with doxorubicin alone [47].

#### 3.1.2. Hyaluronic Acid-Based Nanoparticles for Cancer Therapy

Hyaluronic acid (HA) nanoparticles can encapsulate a wide range of molecules such as small molecule drugs, imaging agents and siRNA for targeted delivery (Table 1). Ganesh et al. developed a HA-based nanoparticle formulation that was able to encapsulate cisplatin, siRNA or near IR (NIR) dye indocyanine green (ICG) for tumor imaging and combinational therapy [48,49]. Due to its anionic nature, HA cannot easily encapsulate the negatively charged siRNA. Therefore, various fatty amines or cationic polyamines were added to the HA backbone to reduce the negative charge density; then the conjugates were used to encapsulate siRNA. siRNA encapsulated in this way showed selective uptake by solid tumor cell models (breast cancer MDA-MB468 cells) and metastatic tumors in mice. However, the in vitro gene-silencing activity results were not exactly translated into mice models [48]. HA nanoparticles were modified and further investigated for reducing cisplatin resistance. ICG was encapsulated by the formulation for whole-body imaging; while cisplatin and siRNA were loaded into the matrix for targeting CD44 receptors in the mice model bearing human lung tumors resistant to cisplatin. Results showed promising efficacy in combination treatments against resistant cancer [49]. Zhone et al. also developed a HA-based nanoparticle for targeted delivery of doxorubicin in a drug-resistant CD44 overexpressed tumor model. HA was functionalized with L-lysine methyl ester and lipoic acid. Then, the HA-conjugates were crosslinked to form the nanoparticles. The release of doxorubicin from the nanoparticles was triggered by glutathione (GSH), which is represented in the cytoplasm. The formulation showed strong targeting ability and antitumor activity toward CD44 receptor-overexpressing doxorubicin-resistant breast cancer cells (MCF-7/ADR cells). Doxorubicin-loaded nanoparticles had high accumulation in the tumors of MCF-7/ADR tumor xenografted nude mice. The tumor growth was inhibited, and the survival time of mice was prolonged [50]. Other HA-based nanoparticles have also been developed for doxorubicin delivery. Yan et al. conjugated a cationic amphiphilic copolymer consisting of PEGylated cationic quaternary amine and *n*-octyl acrylate segments with HA to prepare the nanoparticles. The cationic copolymer was used to reduce the negative charge of the whole system and facilitate cell endocytosis. The acidity of the endosome helped the release of doxorubicin from the vehicle. The drug delivery vehicle showed certain antibacterial ability. In addition to the delivery of doxorubicin, the DDS showed the potential to overcome the bacteria-induced tumor resistance [51]. Dual-functionalized HA nanoparticles to deliver doxorubicin were prepared by Tian et al. HA was modified by a liver targeting ligand, glycyrrhetinic acid and pH-responsive L-histidine. The release of doxorubicin from the system was boosted at lower pH environments because the imidazole groups of histidine were protonated under acidic conditions, resulting in particle size increase. The cytotoxicity of the formulation was evaluated in human hepatoma cell line HepG2 and H22 tumor-bearing mice. The targeted formulations were found to be more effective against tumors than the free drug [52]. Han et al. prepared a formulation for controlled release and targeted delivery of doxorubicin to the nuclei of squamous cell carcinoma cells (SCC7 cells). HA was crosslinked with 2-(Pyridyldithio)-ethylamine (PDA) and polycaprolactone (PCL) to form the nanoparticles. HA was responsible for targeting the CD44-ligand receptors overexpressing in the cancer cells; PDA was a disulfide crosslinker for controlled release of the therapeutic agent; and the hydrophobic PCL facilitated the encapsulation of doxorubicin. These crosslinkages were degraded and caused the intracellular release of doxorubicin from the delivery system in the presence of GSH. The in vitro and in vivo therapeutic performance of doxorubicin was improved by employing this targeted nanoparticle [53]. Cadete et al. prepared docetaxel-loaded HA nanoparticles by a self-emulsification process without using organic solvents or heat. Sodium hyaluronate was functionalized by conjugating with dodecylamide to form an amphiphilic HA derivative, which could avoid the use of cationic surfactants during nanoparticle preparation. Therefore, the safety profile of the delivery system could be improved. This formulation was able to inhibit the growth of A549 cells, and intracellular uptake by cancer cells occurred. The structure of the nanoparticles was stable in human plasma during 24 h of treatment. [54]. Due to the targeting ability of HA, it can serve not only as a carrier in DDSs, but also as a targeting ligand in other nanoparticle systems. Zhang et al. employed HA to coat the surface of poly(lactic-co-glycolic acid) (PLGA) nanoparticles for codelivery of docetaxel and α-naphthoflavone. First, the core of the nanoparticle was modified with polyethyleneimine (PEI) to induce a positive charge. Then, the nanoparticles were coated with HA enhancing cellular uptake of payloads via HA mediated endocytosis. The formulation overcame the multidrug resistance in breast cancer cells (MCF-7/1B1 cells), reversing multidrug resistance and inducing cell apoptosis. In pharmacokinetics study, the bioavailability of docetaxel was significantly enhanced in the HA-coated nanoparticles system as compared to the free form of docetaxel or docetaxel-loaded PLGA nanoparticles [55]. Other HA-coated inorganic nanoparticles, including gold nanoparticles (AuNPs), silica nanoparticles (SiNPs) and metal–organic frameworks (MOF), were also employed for targeted delivery for cancer treatment. Using HA in a DDS helps controlled release, targeted delivery, formulation stabilization, diagnostic imaging and biocompatibility improvement [56,57,58,59,60,61].

#### 3.1.3. Alginate-Based Nanoparticles for Cancer Therapy

Alginate is also employed for the development of nanoparticle DDSs (Table 1). A pH-sensitive and reduction-responsive nanoparticle composed of alginate derivative was reported by Chiu et al. Sodium alginate was firstly thiolated to yield disulfide derivative nanoparticles. Positively charged fluorescein-labeled wheat germ agglutinin (fWGA) was then conjugated to the surfaces of the nanoparticles for targeted delivery of docetaxel to treat colon cancer cells (HT-29 cells). The formulation showed selective uptake by and stronger cytotoxicity toward HT-29 cells but not toward normal mouse fibroblast cells (L929 cells) [62]. It was reported that sodium alginate is not sensitive to enzymatic and hydrolytic degradation in the upper gastrointestinal tract. Therefore, the degradation of encapsulated drugs can be avoided [63]. Disulfide nanoparticles were degraded by GSH overexpressing in cancerous cells, resulting in controlled release at the targeting site or in stimulated gastrointestinal media [62]. A similar controlled release strategy was employed for the delivery of doxorubicin and paclitaxel. Gao et al. employed disulfide crosslinked alginate nanoparticles to encapsulate doxorubicin for cancer treatment. The drug-loaded nanoparticles showed notable inhibition to the HepG2 cells and HeLa cells, while they improved the growth of human liver normal cells (L-O2). The formulation also showed selective uptake within cancer cells. More importantly, the cardiotoxicity of doxorubicin to treat zebrafish was reduced when the drug was encapsulated in the alginate nanoparticles [64]. Ayub et al. prepared disulfide crosslinked sodium alginate to improve the delivery of paclitaxel. Surfaces of the nanoparticles were modified with two polymers, poly(allylamine hydrochloride) (PAH) and poly(4-styrenesulfonic acid-co-maleic acid) sodium salt (PSSCMA) by a layer-by-layer method to prolong the release of payload. Paclitaxel was selectively taken up by HT-29 cells but not human normal colon cells (CRL 1790 cells), and it induced cancer cell death [65]. Zhang et al. employed alginate nanoparticles as carriers for combination therapy. A hydrophobic photosensitizer, pheophorbide A, was conjugated to alginate via disulfide linkage to yield a nanoparticle system in an aqueous environment. Doxorubicin was then loaded onto the nanoparticles for combination therapy. A GSH dose-dependent release manner of doxorubicin and pheophorbide A from the nanoparticles was observed. The nanoparticles transferred both doxorubicin and pheophorbide A into murine melanoma cells (B16 cells) readily. With light irradiation, cytotoxic single oxygen generated by pheophorbide A and doxorubicin showed a stronger antitumor effect as compared to free doxorubicin or nanoparticles without light irradiation. The nanoparticles were preferentially accumulated in the tumor site of B16 tumor-bearing mice with no significant off-target effect. Tumor growth was inhibited by a combination of chemotherapy and photodynamic therapy [66]. There are other similar examples of alginate being employed as a drug carrier for cancer treatment [67]. In some cases, DDSs were composed of alginate and other natural materials such as chitosan to form the nanoparticles. They are discussed in later sections.

#### 3.1.4. Dextran-Based Nanoparticles for Cancer Therapy

Dextran can self-assemble into nanoparticles with various anticancer drugs (Table 1). Thambi et al. reported a carboxymethyl dextran nanoparticle for doxorubicin delivery. Carboxymethyl dextran was conjugated with lithocholic acid via a disulfide linkage for nanoparticle preparation. Doxorubicin was then encapsulated into the nanoparticles. Making use of the disulfide bond, drug release from the nanoparticle’s matrix was accelerated by the presence of GSH. These bioreducible carboxymethyl dextran nanoparticles displayed higher toxicity to SCC7 cells as compared to the doxorubicin-loaded nanoparticles without disulfide linkage. Doxorubicin was effectively delivered to the nuclei of SCC7 cells. The DDS delivered doxorubicin via the enhanced permeation and retention (EPR) effect in a tumor-bearing mice model. They also showed better therapeutic efficacy and biodistribution as compared to simple doxorubicin-loaded nanoparticles [68]. Curcumin is a polyphenol moiety which is able to form conjugates with different polymers [69,70]. It can also be used as a therapeutic agent to treat a wide variety of cancers [71]. Curio et al. synthesized a dextran–curcumin conjugate and allowed the conjugates to form nanoparticles in water media via self-assembly. The dextran–curcumin nanoparticles were employed to encapsulate methotrexate for breast cancer therapy. Methotrexate was released in a sustained manner from the delivery vehicle, while the internalization of the nanoparticles by MCF-7 cells was rapid. Moreover, methotrexate, along with the curcumin moieties, showed a synergistic effect to treat MCF-7 cells. This study gave insights into the combination therapy of employing drug–polymer conjugates as drug carriers to deliver therapeutic agents for cancer treatment [72]. Dextran nanoparticles were prepared to encapsulate chlorin e6 for photodynamic therapy of cancer by Lee et al. Gold nanoparticles were employed as an outer layer to increase the stability of the dextran-based nanoparticles. The whole system was very stable even in serum for six days. The amount of singlet oxygen generated by the metallic-polymeric nanoparticles system was not as much as that of free chlorin e6 or chlorin e6-loaded dextran nanoparticles in vitro. However, the nanoparticles were taken up efficiently by SCC7 cells regardless of the existence of gold deposition. In vivo imaging found that the excellent stability of gold-coated nanoparticle protected chlorin e6 delivery to tumor sites without leakage. Antitumor activity was shown because a sufficient amount of chlorin e6 accumulated at the tumor sites; this amount was much higher than that of free chlorin e6 or chlorin e6-loaded dextran nanoparticles without gold coating [73]. A lipid-modified dextran-based polymeric nanoparticle formulation was designed by Zhang et al. for the encapsulation of microRNAs to treat osteosarcoma cells (KHOS cells and U-2OS cells). Dextran acrylate was modified by stearyl amine in order to achieve self-assembly of nanoparticles. After loading the microRNAs into the nanoparticles, the stability of the microRNAs was greatly improved. The dextran nanoparticles could effectively deliver microRNAs into carcinoma cells for transfection, resulting in suppression of osteosarcoma cell proliferation and growth [74]. PEGylated dextran nanoparticles were prepared by Foerster et al. to target myeloid cells of the liver. Properties of PEGylated nanoparticles and non-PEGylated nanoparticles for siRNA delivery were compared. PEGylation of the dextran nanoparticles prevented aggregation of the particles to yield smaller particles. Furthermore, PEGylated nanoparticles could cause changes in biodistribution and cellular uptake without inducing cytotoxicity. PEGylation of the dextran nanoparticles reduced their uptake by peripheral blood mononuclear cells and murine cell lineages in vitro and significantly shifted dextran nanoparticle accumulation from lungs to liver. The low molecular weight PEGylation did not affect the plasma circulation time. The PEGylated dextran nanoparticles were almost cleared within 24 h after administration. Overall, PEGylated dextran nanoparticles showed potential for use in liver macrophage targeting [75]. A dextran nanoparticle with pH-dependent self-assembly manner was developed by Tang et al. Folic acid was conjugated with dextran to yield the nanoparticle system. In the system, folic acid facilitated pH-dependent self-assembly due to its hydrophobic nature and facilitated doxorubicin loading by electrostatic interaction. Folic acid was also responsible for targeting folate receptors overexpressed in cancer cells. The nanoparticle formulation showed the highest tumor inhibition rate both in vitro and in vivo, and the survival time of murine breast carcinoma 4T1 cell subcutaneous tumor-bearing mice was prolonged as compared to free doxorubicin and folic acid blocked nanoparticle system [76].

**Table 1:** Examples of polysaccharides-based nanoparticles.

Materials	Composition of Nanoparticles	Significances	Ref.
Chitosan	Ascorbic acid, pentasodium tripolyphosphate	Antioxidative; reduced viability of cervical cancer cells; nontoxic to human normal cells	[40]
	EGFR binding peptide, PEG2000, Mad2 siRNA	Selective uptake by NSCLC cells; stronger tumor inhibition in a drug-resistant model	[41,42]
	Folate, curcumin	Targeted folate receptors; enhanced toxicity to breast cancer cells; controlled release in acidic environments	[43]
	Glycyrrhetinic acid, doxorubicin	Enhanced cellular uptake and cytotoxicity of doxorubicin	[45]
	PNVCL, cell-penetrating peptide, doxorubicin	Controlled in acidic and hyperpyrexic conditions; selective cellular uptake; stronger tumor inhibition and lower systemic toxicity	[47]
Hyaluronic acid	Cisplatin, siRNA, near IR dye indocyanine green (ICG), various fatty amines or cationic polyamines	Targeted CD44 receptors; effective in combination treatments against resistant cancers	[48,49]
	L-lysine methyl ester, lipoic acid, doxorubicin	Controlled release of doxorubicin triggered by GSH; targeted CD44 receptors	[50]
	PEGylated cationic quaternary amine, *n*-octyl acrylate segments, doxorubicin	Controlled release in acidic environments; antibacterial; overcame bacteria-induced tumor resistance	[51]
	Glycyrrhetinic acid, L-histidine, doxorubicin	Controlled release in acidic environments; improved antitumor efficacy of doxorubicin	[52]
	Polycaprolactone, 2-(Pyridyldithio)-ethylamine, doxorubicin	Improved performance of doxorubicin; targeted delivery; controlled release in acidic environments	[53]
	Dodecylamide, docetaxel	Inhibited the growth of A549 cells; stable in human plasma	[54]
	PLGA, PEI, docetaxel, α-naphthoflavone	Overcame the multidrug resistance; improved bioavailability of docetaxel	[55]
Alginate	Thiolated sodium alginate, fluorescein-labeled wheat germ agglutinin (fWGA), docetaxel	Selective uptake by cancer cells; stronger cytotoxicity toward HT-29 cells; degraded by GSH	[62]
	Disulfide crosslinked alginate, doxorubicin	Improved safety profile of doxorubicin; selective uptake by cancer cells;	[64]
	Poly(allylamine hydrochloride), poly(4-styrenesulfonic acid-co-maleic acid) sodium salt, paclitaxel	Selective uptake by HT-29 cells; induced cell death to the cancer cells	[65]
	pheophorbide A, doxorubicin	GSH dose-dependent release manner of payloads; accumulated in the tumor site; combination of chemotherapy and photodynamic therapy	[66]
Dextran	Carboxymethyl dextran, lithocholic acid, doxorubicin	Release triggered by GSH; improved therapeutic efficacy and biodistribution profile of doxorubicin	[68]
	Curcumin, methotrexate	Sustained release; synergistic effect in treating MCF-7 cells.	[72]
	Chlorin e6, gold nanoparticles	Efficient cellular uptake; no leakage; accumulation of chlorin e6 at tumor site	[73]
	Dextran acrylate, stearyl amine microRNAs	Stabilized and delivered microRNAs into the carcinoma cells; suppressed osteosarcoma cell proliferation	[74]
	PEGylated dextran, siRNA	Changed biodistribution and cellular uptake without affecting cytotoxicity	[75]
	Folic acid, doxorubicin	Enhanced tumor inhibition; targeting folate receptors	[76]

#### 3.1.5. Albumin-Based Nanoparticles for Cancer Therapy

Albumin is the protein-based natural ingredient most commonly used for cancer drug delivery (Table 2). Aljabali et al. employed bovine serum albumin nanoparticles to improve the solubility and bioavailability of piceatannol for colon cancer therapy. The nanoparticle system was stabilized by glutaraldehyde crosslinking and it could be taken up by colon cancer cell lines (CaCo-2 and HT-29 cells) via endocytosis, due to the nanosize effect. The anticancer activity of piceatannol was improved after it was fabricated into the nanoparticle. It downregulated the expression of nuclear p65 and hypoxia-inducible factor-1α in colon cancer cells more effectively than the free piceatannol. The nanoparticles also showed stronger suppression of tumor growth in the murine model of chemically induced colon cancer [77]. Similar results were reported previously, when incorporating other natural compounds such as curcumin or caffeic acid phenethyl ester into polymeric nanoparticles consisting of albumin or synthetic polymer [78,79]. Another bovine serum albumin nanoparticle was prepared to encapsulate curcumin by Jithan et al. for the treatment of breast cancer. The dissolution rate and solubility of curcumin were improved by the DDS. The nanoparticle system provided sustained release of curcumin both in vitro and in vivo. As compared to free curcumin, DDS-delivered curcumin showed stronger antitumor activity to treat the breast cancer cell line (MDA-MB-231 cells). Higher intracellular drug levels with the drug-loaded nanoparticles were found. The bioavailability of curcumin was improved by administering it in albumin nanoparticles, proven by pharmacokinetics study in rats [80]. Motevalli et al. employed bovine serum albumin nanoparticles to encapsulate curcumin and doxorubicin for blocking the adaptive treatment tolerance (ATT) of MCF-7-resistant breast cancer cells. The release of drugs from the nanoparticles was assessed in an acidic buffer that simulated the lysosome microenvironment. Both drugs were released from the nanoparticles with first-order kinetics. Curcumin showed slower release as compared to doxorubicin, presumably because it is more hydrophobic than doxorubicin. Therefore, curcumin could interact with the domains of albumin more strongly [81,82]. Experimental results exhibited that curcumin and doxorubicin coencapsulated in albumin nanoparticles were able to decrease the ATT effect. On the contrary, if curcumin and doxorubicin were delivered on separate nanoparticles, curcumin would aggregate and be entrapped by lysosomes, making it unable to block P-glycoprotein in the cytosol [83]. As a result, any doxorubicin taken up by the cancer cells would be moved out [81]. Another DDS based on bovine serum albumin nanoparticles for codelivery of doxorubicin and cyclopamine was developed by Lu et al. Similar to curcumin, cyclopamine can increase doxorubicin accumulation by inhibiting P-glycoprotein expression [84,85]. Antitumor activity of the formulation was evaluated in doxorubicin-resistant breast cancer cells (MDA-MB-231 cells) and tumor-bearing mice. The nanoformulation significantly reversed the drug resistance in the cancer cell model. It could distribute at the tumor sites and metastatic lymph nodes in the xenograft model, inhibiting tumor growth and reducing distant metastasis [84]. Human serum albumin was also employed for the development of nanoparticle-based DDSs. Onafuye et al. encapsulated doxorubicin in human serum albumin in order to overcome the transporter-mediated drug resistance of cancer cells. Doxorubicin is a substrate of ABCB 1 (P-glycoprotein). A neuroblastoma cell line (UKF-NB-3) and its ABCB1-expressing sublines adapted to vincristine (UKF-NB-3^r^VCR^1^) and doxorubicin (UKF-NB-3^r^DOX^20^) were employed to evaluate the drug-loaded nanoparticles. The albumin-based nanoparticles increased the anticancer activity of doxorubicin in the drug-resistant cell lines, and the UKF-NB-3^r^VCR^1^ cells were resensitized to the level of UKF-NB-3 cells [86]. The use of albumin as a nanocarrier seems to improve the transporter-mediated drug resistance as compared to a previous report using synthetic polymer PLGA and polylactic acid (PLA) as the nanocarrier [87]. Kimura et al. prepared doxorubicin-loaded human serum albumin nanoparticles for evaluation of the antitumor effect. 2D and 3D of colon 26 cell cultures and tumor-bearing mice were employed. The cytotoxicity of the nanoformulation was weaker than that of free doxorubicin in vitro, while opposite results were observed in vivo even though there was no significant difference in the biodistribution profiles. Furthermore, the DDS helped suppress tumor metastasis. However, more studies are needed to investigate the mechanisms of how nanoparticles work in treating the cancer cell both in vitro and in vivo [88]. Zhang et al. employed human serum albumin nanoparticles to encapsulate the doxorubicin prodrug. The nanoparticle system was pH-sensitive and aggregated at an acidic environment due to the protonation of carboxylic groups of doxorubicin prodrug, which facilitated tumor accumulation and retention. The prodrug nanoparticles showed stronger cellular uptake and cytotoxicity as compared to doxorubicin nanoparticles while the anticancer efficacy of prodrug nanoparticles was similar to that of doxorubicin nanoparticles in vivo. Both nanoparticles showed lower cardiotoxicity as compared to the free form of doxorubicin [89]. Wang et al. encapsulated a potent cytotoxic derivative of maytansine. The safety profile of the derivative in an animal model was improved. The release profiles could also be adjusted by tuning the ratio of drug to albumin during nanoparticle preparation. After administration, the formulation protected the drug molecules from premature release, and rapid body clearance was avoided [90]. Paclitaxel could also be encapsulated in human albumin nanoparticles. PEGylated albumin nanoparticles were developed by Lee et al. to improve the therapeutic performance of paclitaxel for cancer chemotherapy. This formulation was lyophilized and rehydrated prior to use. Therapeutic efficacy was compared with the commercial drug, Abraxane^®^ in various breast cancer cell lines. The paclitaxel formulation even exhibited a stronger cytotoxic effect. In a tumor-bearing mice model, PEGylated nanoparticles prolonged the circulation time of paclitaxel more than 96 h and paclitaxel was able to accumulate in the tumor, resulting in an outstanding antitumor effect and improving the survival rate [91]. Doxorubicin-loaded albumin nanoparticles carrying positive charge were prepared by Abbasi et al. for breast cancer treatment [92]. Polyethyleneimine was employed to induce a positive charge and stabilize the nanoparticles [93]. The addition of the cationic polymer improved the cellular uptake by MCF-7 cells, and the formulation showed stronger cytotoxicity in breast cancer therapy than free doxorubicin [92].

#### 3.1.6. Gelatin-Based Nanoparticles for Cancer Therapy

Gelatin can be employed as a drug carrier to treat a wide range of cancers (Table 2). Lu et al. developed gelatin nanoparticles of paclitaxel for intravesical bladder cancer treatment. DDS was used to protect paclitaxel from being diluted by urine production, leading to therapeutic failure. The release of paclitaxel from the gelatin nanoparticles was rate-limited by the drug solubility in the medium. Therefore, the concentration of paclitaxel would not change no matter the volume of urine. In the dog model, the systemic absorption of the nanoparticles was low; the drug targeted and accumulated in bladder tissues, with pharmacologically active concentration for at least one week [94]. Surface-modified gelatin nanoparticles were designed by Wang et al. for targeted doxorubicin delivery. A targeting ligand, 3-carboxyphenylboronic acid (3-CPBA), was used to decorate the surface of the gelatin nanoparticles. The ligand was employed to target sialic acid which is overexpressed in tumor cells. The formulation was stable, and the release rate of doxorubicin from the nanoparticles increased significantly in the acidic environment. The 3-CPBA promoted cell penetration of the nanoparticles. Doxorubicin was internalized by tumor cells, and accumulated in the cell nuclei. The 3-CPBA-modified gelatin nanoparticles showed the highest tumor accumulation and the strongest antitumor activity in the H22 tumor-bearing mice as compared to free drug or conventional gelatin nanoparticles [95]. Hu et al. designed a shrinkable nanoparticle DDS by using gelatin and dendritic poly-L-lysine (DGL) [96]. DGL is a dendrimer (about 5 nm) which has strong penetrability into tumors [97]; while gelatin can be biodegraded by gelatinases including MMP-2, which is highly expressed in tumors [98]. Doxorubicin was firstly conjugated with DGL, and then decorated onto the gelatin nanoparticles. The whole system was retained in the tumor via the EPR effect. Then, MMP-2 present in the tumor microenvironment hydrolyzed the gelatin nanoparticles to release the small doxorubicin/DGL conjugates, which facilitated the deep penetration of doxorubicin into the core of tumor issues [96]. Also, MMP-2-responsive gelatin nanoparticles for codelivery of doxorubicin and 5-aminolevulinic acid (5-ALA) were developed by Xu et al.; 5-ALA is a photosensitizer for photodynamic therapy. The release of payloads from the nanoparticles was proven to be triggered by the presence of MMP-2 in the tumor microenvironment. Synergistic effects from chemotherapy and photodynamic therapy were achieved when inducting laser irradiation both in vitro and in vivo. Furthermore, the gelatin nanoparticles did not show obvious cardiotoxicity as compared to free doxorubicin in S180 sarcoma cell-bearing mice [99]. Employing gelatin nanoparticles for drug delivery to treat NSCLC was reported. Karthikeyan et al. encapsulated resveratrol in gelatin nanoparticles and evaluated its therapeutic efficacy in NCI-H460 lung cancer cells. In vitro release study showed that sustained release of resveratrol from the nanoparticles was achieved after an initial burst release. The cellular uptake of the formulation was rapid. The resveratrol nanoparticles showed stronger antitumor activity in the lung cancer cells and Swiss albino mice as compared to the free drug [100,101]. Kuo et al. modified the surface of gelatin nanoparticles with phytohemagglutinin erythroagglutinating (PHA-E) for targeted delivery of gemcitabine to treat NSCLC cells. PHA-E was able to target and inhibit EGFR overexpressed in the tumor microenvironment. Briefly, gelatin was conjugated with NeutrAvidin-tetramethylrhodamine to yield fluorescent gelatin nanoparticles for tracking. PHA-E and gemcitabine were grafted to the nanoparticles to form the DDS. The targeting nanoparticles could inhibit the growth of NSCLC cells A549 and H292 by mediating EGFR phosphorylation and causing cell apoptosis [102]. Another DDS using magnetic gelatin nanoparticles for targeted delivery of gemcitabine was designed by Sanlier et al. The magnetic gelatin nanoparticles were synthesized by mixing gelatin with an iron oxide suspension, and then gemcitabine was physically adsorbed onto the magnetic nanoparticles. Release of gemcitabine from the nanoparticles was pH-dependent and controllable. Sustained release was achieved as compared to the free drug [103]. Gemcitabine could also be delivered by gelatin nanoparticles for pancreatic cancer treatment. Xu et al. developed ERFR-targeting thiolated gelatin nanoparticles to deliver wt-p53 plasmid or gemcitabine. Both gelatin nanoparticle formulations showed efficient antitumor activity in human pancreatic adenocarcinoma (Panc-1)-bearing mice. When the two nanoparticle formulations were used together, therapeutic effects were more outstanding than those of single anticancer agents [104,105].

**Table 2:** Examples of protein-based nanoparticles.

Materials	Composition of Nanoparticles	Significances	Ref.
Albumin	Bovine serum albumin, piceatannol, glutaraldehyde	Improved anticancer activity of piceatannol	[77]
	Bovine serum albumin, curcumin	Enhanced dissolution rate, solubility, bioavailability and antitumor activity of curcumin	[80]
	Bovine serum albumin, curcumin, doxorubicin	Controlled release in an acidic environment; decreased the adaptive treatment tolerance effect	[81]
	Bovine serum albumin, doxorubicin, cyclopamine	Reversed drug resistance in cancer cells model; distributed at the tumor sites; reduced distant metastasis	[84]
	Human serum albumin, doxorubicin	Increased the anticancer activity of doxorubicin in the drug-resistant cell lines	[86]
	Human serum albumin, doxorubicin	Weaker cytotoxicity in vitro; opposite in vivo results; suppressed tumor metastasis	[88]
	Human serum albumin, doxorubicin prodrug	Stronger cellular uptake; improved cytotoxicity in vitro; lower cardiotoxicity	[89]
	Human serum albumin, derivative of maytansine	Improved safety profile; controllable release; protected drug molecules from body clearance	[90]
	Human serum albumin, paclitaxel	Could be lyophilized and rehydrated prior to use; better therapeutic efficacy than Abraxane^®^; prolonged the circulation time	[91]
	Human serum albumin, PEI, morphogenetic protein-2	Improved cellular uptake and cytotoxicity in breast cancer therapy	[92]
Gelatin	Gelatin, paclitaxel	Protected paclitaxel from dilution by urine; drug targeted and accumulated in bladder tissues, with pharmacologically active concentration for at least 1 week	[94]
	Gelatin, doxorubicin, 3-carboxyphenylboronic acid	Controlled release in acidic environments; higher tumor accumulation and antitumor activity	[95]
	Gelatin, dendritic poly-L-lysine, doxorubicin	Hydrolyzed by MMP-2 to release the small doxorubicin/DGL conjugates; facilitated deep penetration of doxorubicin	[96]
	Doxorubicin, 5-ALA	Release triggered by MMP-2; synergistic effects from chemotherapy and photodynamic therapy	[99]
	Gelatin, resveratrol	Sustained release; rapid cellular uptake; improved antitumor activity as compared to the free drug	[100,101]
	Gelatin, phytohemagglutinin erythroagglutinating, gemcitabine	Targeted EGFR; inhibited cancer cell growth by mediating EGFR phosphorylation and causing cell apoptosis	[102]
	Gelatin, iron oxide, gemcitabine	Controllable and pH-dependent release manner; sustained release	[103]
	ERFR-targeted thiolated targeted gelatin, gemcitabine, wt-p53 plasmid	Efficient antitumor activity in human pancreatic adenocarcinoma-bearing mice; synergistic effect for combination therapy	[104,105]

#### 3.1.7. Co-Natural Polymer-Based Nanoparticles for Cancer Therapy

More than one natural material can be employed in one nanoparticle’s DDS (Table 3). Deng et al. developed hyaluronic acid–chitosan nanoparticles for codelivery of microRNA-34a and doxorubicin to treat triple-negative breast cancer. The hyaluronic acid–chitosan nanoparticle (HA-CS NPs) is a polyionic nanocomplex system, consisting of polycations and oppositely charged polyanions. The nanocomplexes were employed to encapsulate positively charged doxorubicin and negatively charged microRNA-34a. Codelivery of doxorubicin and microRNA-34a achieved synergistic tumor inhibition, with a reduction in both drug resistance and side effects of doxorubicin [106]. Shargh et al. developed gelatin–albumin hybrid nanoparticles for breast cancer therapy. Albumin is the most native plasma protein while gelatin was chemically modified by incorporating amine residues to yield a positively charged biopolymer. A tropomyosin receptor kinase A (Trk A) inhibitor, GNF-5837 was encapsulated by the nanocomplexes for drug delivery. The gelatin–albumin hybrid could be biodegraded by MMP-2 to yield small drug-bound albumin for efficient matrix diffusion and cellular uptake. Therapeutic efficacy of the encapsulated GNF-5837 was improved as compared to the free GNF-5835 [107]. Song et al. employed anionic alginate and cationic chitosan to prepare the nanoparticles for the encapsulation of curcumin. These two natural materials were coated on iron oxide nanoparticles layer-by-layer. The release profile of curcumin was controlled by modifying the number of chitosan and alginate layers. With the aid of a magnetic field, targeted delivery of curcumin by the nanoparticle system was achieved. Curcumin-loaded nanoparticles showed stronger cytotoxicity to the breast cancer cell line MDA-MB-231 than breast cancer cell HDF. Therapeutic performance of curcumin was improved as compared to the free drug [108]. Sorasitthiyanukarn et al. also employed alginate/chitosan nanocomplexes to deliver curcumin diglutaric acid (a prodrug of curcumin) for cancer treatment. Incorporation of alginate into chitosan improved the protection and controlled release of encapsulated compounds, especially compared to the single use of chitosan or alginate as the drug carrier. The nanoparticles showed higher in vitro cellular uptake in Caco-2 cells and enhanced anticancer activity in Caco-2, HepG2 and MDA-MB-231 cells [109]. Edelman et al. developed hyaluronic acid-serum albumin conjugate-based nanoparticles for targeted therapy. HA served as a hydrophilic block and as a CD44 targeting ligand. The formulation was selectively internalized by cancer cells overexpressing CD44. Paclitaxel or other hydrophobic drugs could be encapsulated in the nanoparticles. The hyaluronic acid-serum albumin conjugates prevented paclitaxel from aggregation and crystallization. Therapeutic efficacy of the drug was also improved. Drug-loaded nanoparticles were significantly more cytotoxic to cancer cells overexpressing CD44 than to cells lacking CD44, due to selective internalization [110].

Various polymeric nanoparticles and different strategies for cancer drug delivery have been reviewed and discussed in the above paragraphs. To sum up, systemic circulation time can be prolonged by conjugating PEG with the nanoparticles. Despite improving the pharmacokinetics and solubility profile of therapeutic agents, the lack of tumor selectivity also restricted the therapeutic applications. Targeting ability can be enhanced by employing HA as the carrier or attaching targeting ligands such as peptide on the surface of nanoparticles. Inducing positive charge to the system is able to enhance the stability and cellular uptake of the nanoparticles. Stimuli-responsive systems such as pH-sensitive nanoparticles can be obtained by making use of the disulfide crosslinkers. Combination therapy can be achieved if loading more than one therapeutic agent in the nanoparticles. In order to broaden the applications of the mentioned natural polymers in drug delivery, other DDSs are discussed in the next section.

### 3.2. Other Natural Ingredient-Based Drug Delivery Systems

#### 3.2.1. Polymeric Gel

Polymeric gels are polymer-solvent systems in which a three-dimensional network is formed by the crosslinked polymeric matrix; it can entrap a large amount of solvent (10- to 100-times the polymer weight) [111]. Consisting of various types of natural materials, polymeric gels have the advantages of increasing bioavailability, adjustable mechanical strength and thermoreversible gelation properties [25]. Gelatin hydrogel was employed to codeliver tetrandrine- and paclitaxel-loaded nanoparticles for combination cancer therapy. Drugs were coloaded onto polycaprolactone (PCL) nanoparticles. The dual drug-loaded nanoparticles were then encapsulated by crosslinked gelatin to form the hydrogel. When the temperature increased, this gel changed from solid jelly to liquid at 37 °C. Drug-loaded particles were released in a sustained manner due to the phase change of gelatin. Therefore, controlled release at the tumor site could be achieved [112]. Collagen could also form a gel for drug delivery. Watanabe et al. employed collagen as the carrier for the delivery of paclitaxel-loaded hydroxyapatite nanoparticles to treat metastatic cancer cells. Due to the existence of matrix metalloproteinase, collagen was degraded to release the drug-loaded nanoparticles. Before embedding into the collagen gel, the paclitaxel nanoparticles showed lower cytotoxicity than the free drug in the poorly metastatic MCF-7 and highly metastatic MDA-MB-231 breast cancer cells. It was caused by the uneven distribution of hydroxyapatite nanoparticles in the media. Loading the nanoparticles into collagen gel enhanced the antitumor activity of the nanoparticles system [113]. Injectable gel can be composed of alginate. A multifunctional alginate cryogel loaded with acetalated dextran nanoparticles was developed to codeliver antigens for in situ vaccination. Nultin-3a is a gene activator of a tumor suppressor gene, p53. Loading Nultin-3a onto dextran nanoparticles increased its immunoadjuvant properties; while loading the nanoparticles into the cryogel enhanced the accumulation of the payloads in the tumor tissue, resulting in better therapeutic performance [114].

#### 3.2.2. Polymeric Micelles

A micelle is an aggregate of amphiphiles, or surfactants, dispersed in an aqueous medium. Its structure consists of hydrophilic head-groups facing the solvent and hydrophobic tails surrounding the interior core space of the micelle (Figure 2). Micelles are small, in the range of 5–100 nm, and are usually employed for enhancing solubility of drugs, prolonging the circulation time, and prompting passive targeting to a tumor site via EPR effect [115]. Chitosan-based polymeric micelles with pH and light dual response were reported by Meng et al. A light-sensitive agent *o*-nitrobenzyl succinate (NBS), which was grafted onto the main chain of a glycol chitosan to yield a conjugate, which formed micelles via self-assembly. The hydrophobic anticancer drug, camptothecin was encapsulated in the micelles. The drug was released from the micelles rapidly under acidic conditions with UV light irradiation. Limited camptothecin was released if the micelles were only triggered by UV light or low pH. The formulation showed good biocompatibility and was internalized effectively by MCF-7 cells. The DDS achieved a controlled and targeted release without affecting the anticancer activity of camptothecin [116]. Karabasz et al. designed a micelle formulation of sodium alginate-curcumin conjugates. The hydrophobic curcumin steadily conjugated hydrophilic sodium alginate to form amphiphiles, creating stable micelles. The formulation was not toxic to mouse endothelial cells nor to cells isolated from human blood, but it showed strong cytotoxicity to various cancer cell lines. In the mice model, the formulation had no toxicity, but therapeutic effects were not as significant as those in vitro [117,118]. Dextran can also be used to form micelles. Dextran–doxorubicin prodrug micelles were obtained by Jin et al. Dextran hydrazine was coupled with hydrophobic deoxycholic acid and doxorubicin was also linked to the dextran hydrazine. This conjugate formed an acid-labile prodrug micelle. The hydrazine linker between dextran hydrazine and doxorubicin could be hydrolyzed at an acidic endosomal pH to release the doxorubicin. The formulation inhibited the growth of MCF-7 and ovarian cancer cells SKOV-3 in vitro, and it showed good antitumor activity in a SKOV-3 tumor-bearing mice model with low systemic toxicity [119].

#### 3.2.3. Liposomes

Liposomes are spherical vesicles consisting of one or more phospholipid bilayers. They have an aqueous core for the encapsulation of hydrophilic molecules and a lipid bilayer for the loading of lipophilic particles (Figure 2). It is commonly used as a delivery vehicle for targeted cancer treatment [120]. Natural phospholipids can be obtained from vegetables such as soybeans and animal materials such as egg yolk and milk. Phosphatidylcholine (PC) is the main phospholipid component for the preparation of liposomes. Conventional liposomes, PEGylated liposomes, and surface-modified liposomes have already been used to deliver a wide range of therapeutic agents to treat various types of cancer [121,122,123]. Cell membranes are usually composed of phospholipid bilayers similar to liposomes. Therefore, the lipid bilayer of liposomes can also be fused with the biological membranes of erythrocytes, platelets, cancer cells and bacterium which have been disrupted via sonication or nitrogen cavitation for drug delivery [124,125]. Cao et al. developed a macrophage membrane-decorated liposome for lung metastasis of breast cancer targeting. First, emtansine was encapsulated by conventional liposomes. The macrophage membrane was mixed with the emtansine liposome for camouflage. The membrane decoration significantly increased the cellular uptake of liposomes in metastatic 4T1 breast cancer cells and showed inhibition on cell viability. It also enhanced the targeting ability and therapeutic performance of liposomes in the mice model with cancer metastasis [124]. AlQahtani et al. made use of the erythrocyte membrane to coat the 5-fluorouracil-loaded liposomes for liver cancer targeting. The formulation displayed a sustained release profile and a delayed cytotoxic effect to HepG2 cells as compared to free 5-fluorouracil, indicating that properties of liposomes can be controlled by membrane decoration [125]. Outer membrane vesicles (OMVs) are nanosized proteoliposomes derived from the outer membrane of Gram-negative bacteria, which can serve as a DDS for cancer therapy. Kuerban et al. prepared a doxorubicin-loaded OMV for NSCLC treatment. The vesicles exerted intensive cytotoxic effects of A549 cells as the OMVs entered the A549 cells efficiently. The doxorubicin-loaded OMVs also suppressed the tumor growth in A549 tumor-bearing BALB/c nude mice with good tolerability and pharmacokinetic profile as compared to free doxorubicin [126]. When using genetically modified OMVs as a cancer immunotherapeutic agent, it was reported that interferon-γ dependent antitumor effect was induced due to the accumulation of OMVs in the tumor tissue. Results implied that the use of OMVs may be suitable for combination therapy to deliver different therapeutic agents and induce antitumor responses at the same time in the future [127]. Besides the cell membrane, other natural ingredients such as chitosan can also be employed for hybrid liposomes preparation. Peng et al. developed hybrid liposomes composed of amphiphilic chitosan and phospholipid to encapsulate curcumin. The hybrid liposomes showed better ionic and thermal stability and more sustained release as compared to conventional liposomes. The bioavailability of curcumin was also improved [128]. Liposomes can also be used to cloak inorganic or organic cores for different therapeutic purposes. Nam et al. reported a pH-responsive gold nanoparticles-in-liposome hybrid. The PEG-grafted liposomes successfully enhanced systemic circulation and tumor accumulation but did not change the bioactivities of the gold nanoparticles [129].

#### 3.2.4. Cell Membrane-Based Drug Delivery Systems

Various types of cells, cell-secreted extracellular vesicles (EVs) and their membranes have been extensively investigated for cancer therapy in recent years. Autologous cell-based DDSs are endogenous; therefore, immunogenicity and toxicity of the DDSs are significantly reduced. As compared to traditional lipid bilayers, the structures of cell membranes are more complex. Cell membranes usually contain moieties such as proteins, carbohydrates and antigens which are related to biofunctions including intracellular communication, immune defense and site-specific migration. During the preparation of cell membrane-based DDSs, the mentioned moieties are largely retained. As a result, the biofunctions of parent cells are transplanted to the DDSs. Besides, the nature and origin of cell membranes determine their unique properties [130]. For example, red blood cell-derived and immune cell-derived membranes are able to help the DDSs avoid the immune clearance while cancer cell-derived membranes allow DDSs targeting the tumor site by an inherent homotypic binding [131]. Whole cells, EVs and cell-derived membranes can be utilized for drug delivery. Among them, cell-derived membranes reveal the best flexibility to develop tailor-made DDSs. Cell membranes are incorporated with different types of nanoparticles such as gold nanoparticles, polymeric nanoparticles, silica nanoparticles, etc. to form biomimetic nanosystems. Nanoparticles are usually used as cores for cell membrane coatings to form core–shell structures. The properties and functions of the systems can be engineered by choosing appropriate materials [130]. For example, Luk et al. coated doxorubicin-loaded PLGA nanoparticles with red blood cell membranes for lymphoma therapy. With the use of red blood cell membranes, the system showed better immunocompatibility and stronger tumor inhibition as compared to the free drug [132]. Xuan et al. coated mesoporous silica nanocapsules with macrophage cell membranes. Surface proteins on the cell membranes endowed the DDS active targeting ability by recognizing tumor endothelium [133]. Through the combination of nanoparticles with cell-derived membranes, formulations can take advantage of both materials to maximize therapeutic efficacy.

#### 3.2.5. Cyclodextrin Inclusion Complex

Cyclodextrins (CDs) are natural cyclic oligosaccharides that have a cavity for encapsulating various molecules to improve their solubility, bioavailability, stability and permeability. Three natural CDs, α-CD, β-CD and γ-CD can be chemically modified to make them suitable for different therapeutic applications. CDs can form an inclusion complex with the payload without forming any covalent bond [134,135]. CDs can also be polymerized to form CD-based nanoparticles for cancer drug delivery [136,137]. Furthermore, CDs can be grafted onto other natural-ingredient-based DDSs such as nanoparticles or hydrogel for functionalization of the system [138,139].

## 4. Discussion and Conclusions

Natural material-based DDSs have shown promising results as compared to conventional therapies. They offer abundant benefits such as biocompatible, biodegradable, nontoxic and nonimmunogenic, and easy modification of the structure for functionalization [9,10]. When designing DDS formulation by using natural materials for cancer treatment, several strategies can be considered. Different natural materials carry various electronic charges, which are highly associated with the encapsulation of therapeutic agents. Electronic charges can be adjusted by conjugating polyamines with the natural materials [48,51,62,92,106]. Mechanical properties, targeting ability and drug release manner can also be controlled by modifying the structure of natural materials with polyamines, small molecules and targeting ligands. In order to optimize the therapeutic performance of DDSs, more than one natural ingredient can be employed. In some cases, synthetic materials, metallic particles or hybrid liposomes are employed to achieve multiple purposes. Examples and recent advances have been discussed in the above sections. Preliminary results have shown the potential of combining organic and/or inorganic components in one system to maximize the functions of DDSs. It may play an important role in future DDSs development.

However, it is not easy to translate the experimental outcomes into clinical applications. Abraxane^®^ is the only natural material-based nanoparticles approved by the FDA to date for cancer therapy [140]. Although some of the natural materials such as chitosan is identified by the FDA as Generally Recognized as Safe (GRAS) for food, most of them have not been approved by the FDA for cancer drug delivery so far. Natural materials originate from a variety of sources; therefore, achieving a high degree of purity is a great challenge due to batch-to-batch variations [10]. The safety profile of natural ingredients may be affected by the contaminants contained in the molecular matrix [26]. Quality standards of each natural material should be built up in order for clinical applications. Furthermore, some derivatives or crosslinking agents can not completely be degraded by natural enzymes. These issues still have to be solved although such DDSs have shown much promise in preclinical studies.

Apart from the biological issues, there are also technical challenges. It is not easy to scale up from laboratory to industrial production. Production requires special equipment and manufacturing can be costly. The stability of the DDSs is also a concern during production. In vivo evaluation is essential for clinical translation. In some cases, in vitro results and in vivo results show contradictions. It requires more effort to study the disease mechanism in detail. DDSs with more precise functions should be developed to overcome the problems case-by-case. Additionally, there is a huge gap between the patients and animal models when evaluating the therapeutic efficacy. Complete testing and evaluations are needed for all cases before natural material-based DDSs can be transferred from the laboratory bench to the bedside in cancer therapy.

Overall, this review has summarized a variety of natural materials that are used as drug delivery vehicles for cancer therapy. The unique properties of these materials make the development of tailor-made DDS possible for improving the therapeutic effects of drugs. The clinical applications of natural material-based DDS are limited at this moment. Once the clinical translation problem is solved, with the advancement of nanotechnology, employing natural material-based DDSs may become a very useful approach for cancer therapy.

## Figures and Tables

**Table 3:** Examples of co-natural polymer-based nanoparticles.

Materials	Composition of Nanoparticles	Encapsulation Advantages	Ref.
Hyaluronic acid, chitosan	Hyaluronic acid, chitosan, microRNA-34a, doxorubicin	Achieved synergistic effects; reduced drug resistance and side effects of doxorubicin	[106]
Gelatin, albumin	Gelatin, albumin, GNF-5837	Efficient cellular uptake; improved therapeutic efficacy of GNF-5837	[107]
Alginate, chitosan	Alginate, chitosan, curcumin, iron oxide	Controllable release; targeted delivery with the aid of magnetic field; improved therapeutic performance of curcumin	[108]
Alginate, chitosan	Alginate, chitosan, curcumin diglutaric acid	Improved protection and controlled release; enhanced cellular uptake and anticancer activity	[109]
Hyaluronic acid, albumin	hyaluronic acid, albumin, paclitaxel	Prevented paclitaxel from aggregation and crystallization; stronger cytotoxicity due to selective internalization	[110]
